# Cross-border purchasing of cigarettes among smokers in Six Countries of the EUREST-PLUS ITC Europe Surveys

**DOI:** 10.18332/tid/100411

**Published:** 2019-03-07

**Authors:** Pete Driezen, Mary E. Thompson, Geoffrey T. Fong, Tibor Demjén, Yannis Tountas, Antigona C. Trofor, Krzysztof Przewoźniak, Witold A. Zatoński, Esteve Fernández, Ute Mons, Constantine I. Vardavas, on behalf of the EUREST-PLUS consortium

**Affiliations:** 1 Department of Psychology, University of Waterloo (UW), Waterloo, Canada; 2 Department of Statistics & Actuarial Science, University of Waterloo (UW), Waterloo, Canada; 3 Ontario Institute for Cancer Research, Toronto, Canada; 4 School of Public Health and Health Systems, University of Waterloo (UW), Waterloo, Canada; 5 Smoking or Health Hungarian Foundation (SHHF), Budapest, Hungary; 6 National and Kapodistrian University of Athens (UoA), Athens, Greece; 7 University of Medicine and Pharmacy ‘Grigore T. Popa’ Iasi, Iasi, Romania; 8 Aer Pur Romania, Bucharest, Romania; 9 Health Promotion Foundation (HPF), Warsaw, Poland; 10 Maria Skłodowska-Curie Institute-Oncology Center (MSCI), Warsaw, Poland; 11 European Observatory of Health Inequalities, President Stanisław Wojciechowski State University of Applied Sciences, Kalisz, Poland; 12 Catalan Institute of Oncology (ICO), Catalonia, Spain; 13 Bellvitge Biomedical Research Institute (IDIBELL), Catalonia, Spain; 14 School of Medicine and Health Sciences, University of Barcelona, Catalonia, Spain; 15 Cancer Prevention Unit and WHO Collaborating Centre for Tobacco Control, German Cancer Research Center (DKFZ), Heidelberg, Germany; 16 European Network for Smoking and Tobacco Prevention (ENSP), Brussels, Belgium; 17 University of Crete (UoC), Heraklion, Greece

**Keywords:** economics, price, taxation, smoking, Europe

## Abstract

**INTRODUCTION:**

The availability of lower-cost cigarettes in neighboring countries provides price-sensitive smokers with incentives to purchase cheaper out-of-country cigarettes. This study estimates the prevalence of and factors associated with cross-border purchasing of cheaper cigarettes among smokers from Germany, Greece, Hungary, Poland, Romania, and Spain. The prevalence of cross-border purchasing was estimated by residential location, defined as living in regions bordering a lower-price country (where prices were at least €1/pack lower), regions bordering a similar- or higher-price country, and internal non-border regions.

**METHODS:**

Data were from a survey of nationally representative samples of adult smokers (n=6011) from Germany, Greece, Hungary, Poland, Romania, and Spain. The primary outcome was purchasing cheaper out-of-country cigarettes in the previous six months. Residential location was defined using The Nomenclature of Territorial Units for Statistics (NUTS2 in Germany and NUTS3 in the other countries). Multivariable logistic regression tested differences in purchasing cheaper out-of-country cigarettes by country and residential location.

**RESULTS:**

Residential location was associated with purchasing cheaper out-of-country cigarettes in Germany and Poland (p<0.05): 31% of German and 11% of Polish smokers living in regions bordering lower-price countries reported purchasing cheaper out-of-country cigarettes in the previous six months. Smokers living in regions bordering lower-price countries had 4.21 times greater odds of purchasing cheaper out-of-country cigarettes compared to smokers living in non-border regions.

**CONCLUSIONS:**

Overall, only a minority of smokers in the six countries purchased cheaper cigarettes outside their country. However, smokers living in regions bordering countries where cigarettes were at least €1/pack lower than their home country had significantly higher odds of purchasing cheaper out-of-country cigarettes. This effect was especially prominent among German smokers. Tax harmonization policies designed to minimize cross-border price differentials can eliminate lower-priced alternatives for price-sensitive smokers.

## INTRODUCTION

Tobacco taxation provides governments with an effective way to increase the price of cigarettes. The World Health Organization (WHO) Framework Convention on Tobacco Control (FCTC) highlights tax and price policies as the single most important strategy for decreasing tobacco consumption and reducing tobacco-related morbidity and mortality^1^. In addition to ratifying the WHO FCTC, the European Union (EU) has been a leader in tobacco taxation with the implementation of the Council Directive 2011/64/EU on mandated tobacco excise duties and standards^2,3^. Furthermore, the EU Tobacco Products Directive TPD 2014/40/EU contains measures to address cross-border sales and illicit trade^4^. Although taxation policies are designed to reduce tobacco consumption, the availability of lower-cost cigarettes in neighboring jurisdictions provides price-sensitive smokers in the EU with incentives to purchase such alternatives.

Existing research suggests that smokers living in EU Member States (MS) bordering lower-cost jurisdictions more frequently purchase cigarettes outside their country of residence compared to smokers living in non-border regions^5-7^. Using data from the International Tobacco Control European country surveys, Nagelhout et al.^6^ found that in 2007, 13.4% of German smokers and 23.7% of French smokers living in border regions frequently purchased cigarettes outside their countries. Only 4.9% and 4.6% (respectively) of smokers from non-border regions of these countries made such purchases. In the Netherlands, which does not border lower-cost jurisdictions, only 2.4% of smokers reported frequently purchasing cigarettes outside their country. Likewise, data from the 2012 Eurobarometer survey indicate that EU MS having the highest prevalence of cross-border purchases were also MS that bordered lower-cost jurisdictions^7^.

Several sociodemographic factors influence the cross-border purchase of lower-cost cigarettes, including sex^8^, age^6-10^, race^8,10^, income^6,9,10^, and education^6,7,9^. For example, high-income smokers are more likely to purchase lower-cost cigarettes outside their country than low-income smokers^6,9,10^. Given that some subgroups of smokers are more likely to seek out lower-cost cigarettes than others, the availability of such alternatives points to the importance of reducing price differentials across jurisdictions^11^. EU regulations stipulate that MS levy an overall excise tax of at least €90/1000 cigarettes and at least 60% of the weighted average retail selling price^4^. MS that levy an excise tax of €115/1000 cigarettes need not comply with the 60% criterion^4^. In spite of these regulations, price differentials remain. As of July 2016, the average price of a pack of 20 cigarettes from the most popular price category (MPPC) in the 12 ‘new’ EU MS (Bulgaria, Czech Republic, Estonia, Cyprus, Latvia, Lithuania, Hungary, Malta, Poland, Romania, Slovenia, and Slovakia) was €3.25/pack while the average price in the 15 ‘old’ MS was €6.30/pack^12^.

Building on the work of Nagelhout et al.^6^ and Agaku et al.^7^, this study estimates the prevalence of cross-border purchasing of cheaper cigarettes by region of residence among smokers from six European countries: Germany (DE), Greece (GR), Hungary (HU), Poland (PL), Romania (RO), and Spain (ES). Smokers living in regions bordering countries having MPPC pack prices at least €1 lower than their home country were compared to smokers living in regions bordering countries with similar or higher MPPC pack prices as well as smokers living in non-border areas. This study therefore provides more granular estimates of cross-border purchasing than previous studies^6,7^. Given price differentials between EU MS existed in 2016, it is expected that smokers living in regions bordering lower-cost jurisdictions will be more likely to purchase cheaper out-of-country cigarettes.

## METHODS

### Data source

The current study is part of an EC Horizon-2020 funded study entitled European Regulatory Science on Tobacco: Policy implementation to reduce lung diseases (EUREST-PLUS-HCO-06-2015). Data come from Wave 1 of the EUREST-PLUS International Tobacco Control Six European Country Survey (ITC 6E), a prospective longitudinal cohort survey of nationally representative samples of adult smokers aged 18 years and older from DE (n=1003), GR (n=1000), HU (n=1000), PL (n=1006), RO (n=1001), and ES (n=1001). In each country, smokers were randomly selected using a multi-stage cluster sampling design. Geographic strata were defined using the Nomenclature of Territorial Units for Statistics (NUTS) level 1 regions (NUTS1) in Germany and NUTS2 regions in all other countries crossed with degree of urbanization (urban, intermediate, rural). In all countries, 100 clusters, each the size of an enumeration area, were sampled. Clusters were allocated to strata proportionally to the size of the adult population (aged 18 years and older). Households within clusters were selected using a random walk method. Within selected households, one male and one female smoker were randomly selected (if available).

Data were collected using a tablet-based CAPI questionnaire between June 2016 and September 2016. All respondents provided informed consent to participate. The study was approved by the Research Ethics Board of the University of Waterloo, Ontario, Canada and by local ethics boards in the participating countries. Additional details of the survey methods are described elsewhere^13-15^.

### Measures

#### Outcome variables

Purchases made in the previous six months outside respondents’ country of residence were assessed using the questions: ‘How often in the last 6 months have you bought cigarettes from outside your country but inside the EU?’ and ‘How often in the last 6 months have you bought cigarettes outside the EU?’. Possible responses for each question were: ‘Never’, ‘Only once’, ‘A few times’, ‘Many times’, and ‘All of the time’. Responses were classified into ternary indicators of purchasing behaviors: ‘Never’, ‘Only once’, or ‘At least a few times’. These separate indicators were then pooled into a single measure of making at least a few out-of-country purchases in the previous six months.

Irrespective of how frequently respondents made out-of-country purchases, those who did were asked the reason for their purchase. Respondents who reported doing so because ‘it was cheaper’ were classified as having made an out-of-country purchase because it was cheaper while all other respondents were classified as not having done so.

Finally, respondents were asked about the price they paid for their most recent purchase of cigarettes, whether it was a carton purchase, pack purchase, or a purchase of loose roll-your-own (RYO) tobacco. For factory-made (FM) purchases, prices were computed as the price per cigarette, dividing the total price paid for the entire purchase by the total number of cigarettes purchased. Prices for RYO purchases were converted to cigarette equivalents assuming that 1 RYO cigarette contained 0.75 g of loose tobacco^16^. Price per cigarette was converted to price per pack of 20 cigarettes (per cigarette price × 20). All prices were converted to Euros (HU, PL, RO) using historical exchange rates listed on XE Currency Converter (http://www.xe.com) and the median survey date in each country (HU = 2016-07-09, PL = 2016-07-16, RO = 2016-07-13).

Initial pack prices were inspected for outliers. Obvious data entry errors in reported prices were corrected (e.g. pack prices differing by two orders of magnitude such as €500/pack instead of €5/ pack). Where no obvious correction could be made, prices were set to missing if the natural logarithm of price was more than 2.5 standard deviations from the mean of logarithms^17-19^. Overall, 77 prices (of 5737 reported prices, or 1.3%) were corrected due to data entry errors (DE=14, GR=25, HU=0, PL=27, RO=4, ES=1) while 58 (1.0%) were set to missing (DE=5, GR=23, HU=15, PL=6, RO=1, ES=8).

### Residential location

Using the NUTS classification system, a respondent’s region of residence identified whether or not that respondent lived in an area bordering another European country (NUTS2 in DE, NUTS3 in all other countries). Distance to the nearest bordering country was determined as the Euclidean distance from the geographic centroid of the NUTS region to the border of the nearest country. Geospatial data for computing distances were obtained from Eurostat’s GISCO website^20^; distances were computed using the ‘NNJoin’ plugin (nearest neighbour join, Version 1.3.2) of QGIS (Version 2.18).

**Figure 1:**
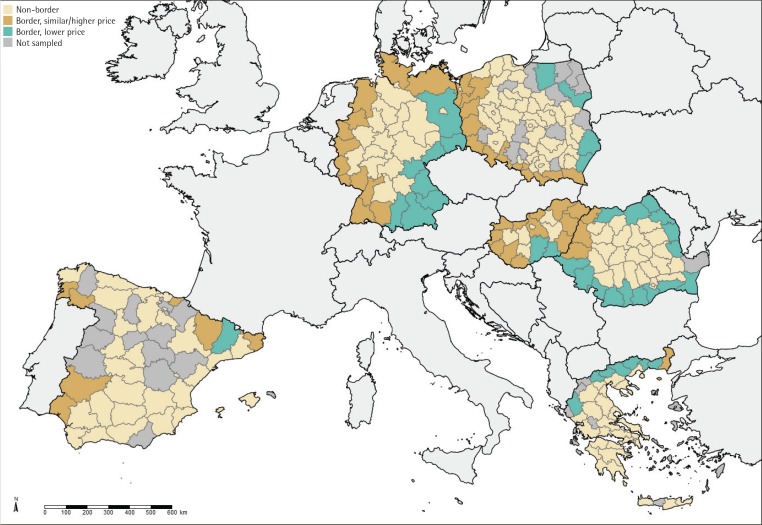
EUREST-PLUS ITC 6E Survey: sampled NUTS regions and cigarette prices in neighbouring countries

For respondents living in a NUTS region bordering another European country, the MPPC pack price was compared between respondents’ country of residence and the nearest neighboring country. Price data, as of July 2016, were obtained from the WHO Report on the Global Tobacco Epidemic^12^. Following Nagelhout et al.6, respondents living in regions where the MPPC pack price in the nearest bordering country was at least €1 lower than the home country price were classified as bordering a lower price country (‘Border, lower price’). Respondents living in regions where the MPPC pack price in the nearest bordering country was within €1 or where prices were higher were classified as bordering a ‘similar/higher’ price country (Supplementary Table 1). Respondents not living in border regions were classified as ‘non-border’ respondents (Figure 1).

### Covariates

Sociodemographic measures were sex (female vs male), age group (25–39, 40–54, ≥55, vs 18–24 years), employment status (employed, otherwise), marital status (not married, married/common-law, windowed/divorced), and degree of urbanization (urban, intermediate, rural). In each country, household income information was collected using local currencies. Different thresholds were used in each country to classify respondents as low, moderate, or high income (Supplementary Table 2). Respondents who refused to provide household income were retained for analysis by including an ‘income not reported’ category. Education was also classified as low, moderate, or high using the International Standard Classification of Education^21^. Low education was defined as ‘pre-primary education/no education’, ‘primary education’, and ‘lower secondary education’. Moderate education was defined as ‘upper secondary education’, ‘post-secondary non-tertiary education’, and ‘short-cycle tertiary education’. High education was defined as ‘bachelor or equivalent’, ‘master or equivalent’, and ‘doctoral or equivalent’.

Relevant behavioral measures were smoking status (daily, weekly, monthly), whether respondents reported smoking RYO cigarettes (exclusively RYO, both RYO and FM, exclusively FM), amount smoked per day (≤10, 11–20, 21–30, ≥31), minutes to first cigarette after waking (≤5, 6–30, 31–60, >60), nicotine dependence (heaviness of smoking index [HSI], coded as 0–2, 3–4, 5–6), and intentions to quit smoking (in the next 6 months, beyond 6 months, no intentions).

### Statistical analysis

Unweighted descriptive statistics described the sociodemographic characteristics of the sample of smokers from each country. Weighted descriptive statistics estimated the percentage of smokers purchasing out-of-country cigarettes by country and residential location. Differences were tested using Wald F tests. Confidence intervals for percentages were estimated using the logit method unless estimated percentages were smaller than 5% or greater than 95% or if estimates were based on unweighted cell counts of 50 observations or fewer. In these cases, exact confidence intervals were estimated^22^.

Logistic regression tested differences in purchasing cheaper out-of-country cigarettes by: a) country, b) residential location, and c) the country × residential location interaction. Linear regression compared self-reported pack prices within countries by: a) purchase of cheaper out-of-country cigarettes and b) exclusive use of RYO cigarettes. All regression models controlled for sociodemographic (sex, age group, income, education, degree of urbanization, employment status) and smoking characteristics (cigarettes smoked/day, time to first cigarette after waking, exclusive RYO use, intentions to quit).

The statistical analysis was conducted using SAS-callable SUDAAN Version 11.0.1 to account for the complex sampling design. Variances were estimated using bootstrap replicate weights (n=500), setting Fay’s adjustment factor^23,24^ to 0.7113249. For all F tests, the denominator degrees of freedom were set to 459 (number of primary sampling units - number of strata).

## RESULTS

### Sociodemographic characteristics of the sample

Sampled smokers recruited in each country differed in several ways (Table 1). While approximately 50% of smokers from Germany were male, a greater percentage of smokers from Greece, Hungary, Poland, and Spain were male. The age distribution of smokers also differed by country: 30% of smokers from Germany, Greece, and Hungary were 55 years or older while less than 10% of smokers were 18–24 years. A greater percentage of smokers from Romania and Spain were under 40 years while 25% were 55 years or older. The majority of smokers were married, ranging from 59% in Germany and Spain to 70% in Romania.

**Table 1:** Characteristics of smokers participating in the EUREST-PLUS ITC 6E Survey (Wave 1; 2016, unweighted)

	*DE (n=1003)*		*GR (n=1000)*		*HU (n=1000)*		*PL (n=1006)*		*RO (n=1001)*		*ES (n=1001)*	
	*(n)*	*%*	*(n)*	*%*	*(n)*	*%*	*(n)*	*%*	*(n)*	*%*	*(n)*	*%*
**Sex**												
Male	(507)	50.5	(544)	54.4	(521)	52.1	(477)	47.4	(581)	58.0	(545)	54.4
Female	(496)	49.5	(456)	45.6	(479)	47.9	(529)	52.6	(420)	42.0	(456)	45.6
**Age group (years)**												
18–24	(88)	8.8	(61)	6.1	(59)	5.9	(72)	7.2	(110)	11.0	(117)	11.7
25–39	(283)	28.2	(255)	25.5	(282)	28.2	(342)	34.0	(300)	30.0	(312)	31.2
40–54	(339)	33.8	(383)	38.3	(357)	35.7	(281)	27.9	(321)	32.1	(323)	32.3
≥55	(293)	29.2	(301)	30.1	(302)	30.2	(311)	30.9	(270)	27.0	(249)	24.9
**Marital status**												
Not married	(249)	24.8	(187)	18.7	(163)	16.3	(189)	19.0	(185)	18.5	(290)	29.0
Married/common-law	(601)	59.9	(695)	69.6	(664)	66.6	(670)	67.4	(704)	70.4	(592)	59.1
Widowed/divorced	(153)	15.3	(116)	11.6	(170)	17.1	(135)	13.6	(111)	11.1	(119)	11.9
**Household income**												
Not reported	(93)	9.3	(197)	19.7	(311)	31.1	(326)	32.4	(59)	5.9	(394)	39.4
Low	(306)	30.5	(180)	18.0	(179)	17.9	(173)	17.2	(226)	22.6	(271)	27.1
Moderate	(347)	34.6	(525)	52.5	(290)	29.0	(353)	35.1	(466)	46.6	(268)	26.8
High	(257)	25.6	(98)	9.8	(220)	22.0	(154)	15.3	(250)	25.0	(68)	6.8
**Education**												
Low	(509)	50.8	(306)	30.7	(617)	61.8	(123)	12.4	(246)	24.9	(410)	41.0
Moderate	(417)	41.7	(488)	48.9	(311)	31.2	(753)	76.1	(629)	63.6	(506)	50.7
High	(75)	7.5	(203)	20.4	(70)	7.0	(114)	11.5	(114)	11.5	(83)	8.3
**Employment status**												
Not employed	(340)	34.0	(422)	42.2	(327)	32.8	(482)	48.2	(436)	43.6	(448)	44.8
Employed	(660)	66.0	(577)	57.8	(671)	67.2	(518)	51.8	(563)	56.4	(552)	55.2
**Residential location**												
Urban	(380)	37.9	(170)	17.0	(342)	34.2	(341)	33.9	(360)	36.0	(532)	53.1
Intermediate	(409)	40.8	(609)	60.9	(368)	36.8	(309)	30.7	(240)	24.0	(320)	32.0
Rural	(214)	21.3	(221)	22.1	(290)	29.0	(356)	35.4	(401)	40.1	(149)	14.9
**Border region**												
Non-border	(488)	48.7	(900)	90.0	(362)	36.2	(700)	69.6	(540)	53.9	(871)	87.0
Border–similar/higher price	(315)	31.4	(10)	1.0	(539)	53.9	(209)	20.8	(80)	8.0	(120)	12.0
Border–lower price	(200)	19.9	(90)	9.0	(99)	9.9	(97)	9.6	(381)	38.1	(10)	1.0
**Smoking status**												
Daily	(883)	88.0	(970)	97.0	(988)	98.8	(962)	95.6	(947)	94.6	(969)	96.8
Weekly	(96)	9.6	(24)	2.4	(9)	0.9	(38)	3.8	(49)	4.9	(19)	1.9
Monthly	(24)	2.4	(6)	0.6	(3)	0.3	(6)	0.6	(5)	0.5	(13)	1.3
**Smokes RYO**												
Exclusive RYO	(108)	10.8	(256)	25.6	(444)	44.4	(70)	7.0	(10)	1.0	(173)	17.3
Both RYO + FM	(150)	15.0	(17)	1.7	(73)	7.3	(118)	11.7	(42)	4.2	(90)	9.0
Exclusive FM	(745)	74.3	(727)	72.7	(483)	48.3	(817)	81.3	(949)	94.8	(738)	73.7

Monthly household income differed by country. The percentage of smokers reporting low income ranged from 17–18% in Greece, Hungary, and Poland to 27% in Spain and 30% in Germany. The percentage of smokers reporting high income ranged from 7% in Spain to 26% in Germany. Twenty per cent of smokers from Greece did not report monthly household income while more than 30% did not report income in the remaining countries, except Germany and Romania.

The majority of smokers from Germany and Hungary were from low education groups while the majority of smokers in the other countries were moderately educated. Most smokers resided in intermediately or highly urbanized areas, although 40% of Romanian smokers were from rural areas. Twenty per cent of German smokers and 40% of Romanian smokers were from areas neighboring a lower-price country. In the remaining countries, fewer than 10% of smokers lived near lower-price countries. The vast majority of smokers in all countries were daily smokers, with the exception of Germany, where 12% were non-daily smokers. With the exception of Hungary, most smokers smoked FM cigarettes alone or in combination with RYO. In Hungary, 44% of smokers exclusively smoked RYO cigarettes.

### Prevalence of cross-border purchasing

Table 2 presents weighted estimates of the prevalence of cross-border cigarette purchasing in each country. More than 90% of smokers from Greece, Hungary, Poland, and Spain never purchased cigarettes outside their country within the previous six months. Just over 10% of smokers from Germany and Romania purchased cigarettes outside their country at least a few times in the previous six months. Nine per cent of German smokers made only one out-of-country purchase in the previous six months. Smokers who purchased out-of-country cigarettes tended to make their purchases from EU MS. Overall, the prevalence of out-of-country cigarette purchases differed by country (all p<0.001). German smokers were more likely than smokers from other countries to report purchasing out-of-country cigarettes because they were cheaper (p<0.001). Almost 15% of German smokers purchased cheaper out-of-country cigarettes compared to 6% of Greek smokers, and 3% of Romanian smokers. Less than 2% of smokers from the remaining countries purchased cheaper out-of-country cigarettes in the previous six months.

**Table 2:** Per cent of smokers in each country who purchased any cigarettes outside their home country in the last six months

*Cigarette purchase*	*DE*			*GR*			*HU*			*Wald F*		
	*(n)*	*%*	*(95% CI)*	*(n)*	*%*	*(95% CI)*	*(n)*	*%*	*(95% CI)*	*F*	*DF*	*p*
**Any purchase outside country**												
Never	(792)	79.2	(75.3–82.6)	(917)	90.6	(86.4–93.6)	(975)	97.7	(96.4–98.6)	13.55	10	<0.001
Only once	(85)	8.9	(6.8–11.6)	(32)	3.0	(2.0–4.3)	(12)	1.1	(0.5–2.1)			
At least a few times	(125)	11.9	(9.2–15.2)	(51)	6.4	(3.9–10.5)	(13)	1.2	(0.6–2.1)			
**Outside home country–inside EU**												
Never	(813)	81.5	(77.7–84.8)	(950)	94.2	(90.8–96.7)	(981)	98.3	(97.2–99.0)	13.30	10	<0.001
Only once	(72)	7.4	(5.4–10.2)	(22)	2.1	(1.3–3.2)	(7)	0.7	(0.3–1.4)			
At least a few times	(117)	11.0	(8.5–14.3)	(28)	3.7	(1.7–6.8)	(12)	1.1	(0.5–1.9)			
**Outside home country–outside EU**												
Never	(944)	94.0	(91.7–95.6)	(947)	94.5	(92.5–95.9)	(986)	98.8	(97.7–99.5)	5.58	10	<0.001
Only once	(34)	3.7	(2.5–5.2)	(18)	1.7	(1.0–2.7)	(9)	0.8	(0.3–1.7)			
At least a few times	(25)	2.4	(1.2–4.1)	(35)	3.9	(2.4–5.9)	(5)	0.4	(0.1–1.1)			
**Outside purchase was cheaper**												
No outside purchase	(856)	85.4	(81.5–88.6)	(952)	94.0	(89.7–96.8)	(991)	99.1	(98.1–99.6)	12.74	5	<0.001
Outside was cheaper	(146)	14.6	(11.4–18.5)	(48)	6.0	(3.2–10.3)	(9)	0.9	(0.4–1.9)			

***Cigarette purchase***	***PL***			***RO***			***ES***			***Wald F***		
	***(n)***	***%***	***(95% CI)***	***(n)***	***%***	***(95% CI)***	***(n)***	***%***	***(95% CI)***	***F***	***DF***	***p***

**Any purchase outside country**												
Never	(935)	92.2	(89.7–94.2)	(890)	87.0	(82.6–90.4)	(948)	95.5	(94.0–96.7)	13.55	10	<0.001
Only once	(22)	2.4	(1.5–3.6)	(18)	2.4	(1.2–4.2)	(26)	2.0	(1.2–3.1)			
At least a few times	(45)	5.4	(3.6–7.7)	(90)	10.6	(7.8–14.3)	(27)	2.5	(1.5–3.9)			
**Outside home country–inside EU**												
Never	(945)	93.1	(90.7–94.9)	(909)	89.4	(86.3–92.0)	(966)	97.4	(96.2–98.3)	13.30	10	<0.001
Only once	(22)	3.0	(1.9–4.4)	(15)	1.8	(0.9–3.4)	(15)	1.1	(0.6–2.0)			
At least a few times	(36)	4.0	(2.6–5.8)	(74)	8.7	(6.7–11.3)	(20)	1.5	(0.8–2.5)			
**Outside home country–outside EU**												
Never	(969)	96.0	(93.9–97.5)	(958)	95.0	(91.4–97.5)	(976)	97.5	(96.1–98.5)	5.58	10	<0.001
Only once	(13)	1.3	(0.6–2.4)	(9)	1.1	(0.4–2.6)	(14)	1.2	(0.6–2.1)			
At least a few times	(22)	2.7	(1.4–4.8)	(33)	3.9	(1.8–7.1)	(11)	1.3	(0.6–2.7)			
**Outside purchase was cheaper**												
No outside purchase	(985)	98.3	(96.8–99.2)	(969)	96.6	(94.1–98.3)	(984)	98.8	(97.9–99.4)	12.74	5	<0.001
Outside was cheaper	(17)	1.7	(0.8–3.2)	(29)	3.4	(1.7–5.9)	(17)	1.2	(0.6–2.1)			

Supplementary Table 3 elaborates these findings, illustrating the frequency with which smokers purchased any cigarettes outside their country, irrespective of whether or not the purchase was made from an EU MS. In most countries, the majority of smokers purchasing outside the country only did so ‘a few times’; it was rare for smokers to purchase cigarettes outside their home country ‘all the time’. For example, only 2.5% of German smokers reported purchasing cigarettes outside Germany all the time. These findings suggest the overall share of out-of-country purchases is likely small in each of the six countries.

Table 3 presents the prevalence of purchasing cheaper out-of-country cigarettes stratified by residential location. In both Hungary and Romania, residential location was not associated with purchasing cheaper cigarettes outside the country (p=0.374 and p=0.394, respectively). In Poland and Germany, however, there was an association between residential location and purchasing cheaper cigarettes outside the country. Eleven per cent of Polish smokers living in regions bordering lower-priced countries purchased cheaper out-of-country cigarettes compared to less than 1% of Polish smokers living in non-border regions (p=0.016). An even greater percentage of German smokers living in regions bordering lower-priced countries purchased cheaper out-of-country cigarettes (31% vs 13% living in non-border regions).

**Table 3:** Per cent of smokers who purchased cheaper cigarettes outside their home country by region of residence

*Purchased cheaper cigarettes outside country*	*Non-border*			*Border–similar/higher price*			*Border–lower price*			*Wald F Test*		
	*(n)*	*%*	*(95% CI)*	*(n)*	*%*	*(95% CI)*	*(n)*	*%*	*(95% CI)*	*F*	*DF*	*p*
**Germany**												
Yes	(67)	13.1	(8.9–19.0)	(22)	7.6	(3.7–13.4)	(57)	31.3	(22.3–41.9)	7.48	2	<0.001
No	(420)	86.9	(81.0–91.1)	(293)	92.4	(86.6–96.3)	(143)	68.7	(58.1–77.7)			
**Greece**												
Yes	(26)	3.2	(2.0–4.8)	(3)	34.1	(8.8–68.9)	(19)	23.3	(5.1–54.2)	2.42	2	0.090
No	(874)	96.8	(95.2–98.0)	(7)	65.9	(31.1–91.2)	(71)	76.7	(45.8–94.9)			
**Hungary**												
Yes	(1)	0.4	(0.0–1.9)	(7)	1.3	(0.4–3.0)	(1)	1.0	(0.0–5.6)	0.98	2	0.374
No	(361)	99.6	(98.1–100.0)	(532)	98.7	(97.0–99.6)	(98)	99.0	(94.4–100.0)			
Poland												
Yes	(6)	0.8	(0.2–2.1)	(0)	0.0		(11)	11.1	(4.6–21.6)	4.15	2	0.016
No	(692)	99.2	(97.9–99.8)	(208)	100.0		(85)	88.9	(78.4–95.4)			
**Romania**												
Yes	(12)	2.5	(0.9–5.3)	(4)	10.1	(2.4–25.3)	(13)	3.3	(0.8–8.8)	0.93	2	0.394
No	(528)	97.5	(94.7–99.1)	(74)	89.9	(74.7–97.6)	(367)	96.7	(91.2–99.2)			
**Spain**												
Yes	(15)	1.2	(0.6–2.2)	(1)	0.6	(0.0–4.2)	(1)	7.4	(0.1–41.3)	0.97	2	0.380
No	(856)	98.8	(97.8–99.4)	(119)	99.4	(95.8–100.0)	(9)	92.6	(58.7–99.9)			

Multivariable logistic regression tested the overall effect of residential location on purchasing cheaper out-of-country cigarettes controlling for sociodemographic factors and smoking behaviors (Table 4). The difference in the prevalence of purchasing cheaper cigarettes between countries is reflected in the magnitude of the odds ratio for German smokers relative to Spanish smokers. Controlling for other factors, German smokers had 9.^22^ times greater odds of purchasing cheaper out-of-country cigarettes compared to Spanish smokers. Consistent with bivariate results in Table 2, Greek smokers also had significantly greater odds of purchasing cheaper out-of-country cigarettes (OR=3.49, 95% CI: 1.77– 6.88). When the effect of residential location was pooled across countries, smokers living in regions bordering lower-priced countries had 4.^21^ times the odds of purchasing cheaper out-of-country cigarettes compared to smokers living in non-border regions (95% CI: 2.39–7.42).

**Table 4:** Odds of smokers purchasing cheaper cigarettes outside their home country (n=5928)

*Covariate*	*OR*	*(95% CI)*	*F Test*	*DF*	*p*
**Sex** (vs male)					
Female	0.88	(0.69–1.12)	1.09	1	0.296
**Age group** (vs 18–24 years)					
≥55	0.94	(0.52–1.72)	0.43	3	0.729
40–54	0.97	(0.57–1.67)			
25–39	1.16	(0.65–2.08)			
**Income** (vs high)					
Not reported	0.69	(0.37–1.27)	2.06	3	0.104
Low income	1.35	(0.84–2.18)			
Moderate income	1.10	(0.72–1.68)			
**Education** (vs high)					
Low	1.36	(0.68–2.73)	0.63	2	0.533
Moderate	1.45	(0.75–2.83)			
**Urban** (vs rural)					
Urban	1.50	(0.81–2.78)	1.19	2	0.305
Intermediate	1.48	(0.87–2.52)			
**Employed** (vs not employed)					
Employed	0.87	(0.62–1.22)	0.65	1	0.421
**Cigarettes/day** (vs ≤10)					
≥31	2.24	(1.10–4.58)	3.46	3	0.016
21–30	1.84	(1.01–3.36)			
11–20	1.90	(1.28–2.83)			
**Minutes to first cigarette** (vs >60)					
Within 5	1.45	(0.79–2.66)	1.94	3	0.123
6–30	1.61	(0.94–2.76)			
31–60	2.01	(1.12–3.62)			
**Smokes RYO** (vs exclusive FM)					
Exclusive RYO	1.72	(1.09–2.72)	5.42	2	0.005
Both RYO + FM	2.04	(1.26–3.32)			
**Plans to quit** (vs within 6 months)					
No plans to quit	0.71	(0.46–1.11)	1.32	2	0.268
Beyond 6 months	0.85	(0.54–1.34)			
**Country** (vs Spain)					
Germany	9.22	(5.05–6.82)	18.05	5	<0.001
Greece	3.49	(1.77–6.88)			
Hungary	0.55	(0.21–1.45)			
Poland	1.14	(0.49–2.65)			
Romania	1.44	(0.58–3.60)			
**Border region** (vs non-border region)					
Border–lower	4.21	(2.39–7.42)	16.05	2	<0.001
Border–similar/higher	0.85	(0.46–1.55)			

Daily cigarette consumption was strongly associated with purchasing cheaper cigarettes (p=0.016). Compared to lighter smokers, those who smoked >30 cigarettes/day had 2.24 times the odds of purchasing cheaper out-of-country cigarettes (95% CI: 1.10– 4.58). Similar effects were seen among smokers consuming between 11 and 30 cigarettes per day. Time to first cigarette (TTF) was not associated with purchasing cheaper cigarettes. A separate model (results not shown) using HSI instead of CPD + TTF showed that HSI was not significantly related to purchasing cheaper out-of-country cigarettes (F=2.20; df=2, 459; p=0.11).

Interestingly, RYO smokers had greater odds of purchasing cheaper out-of-country cigarettes than FM smokers. Exclusive RYO smokers had 1.72 times greater odds compared to FM smokers while those who smoked both RYO and FM cigarettes had 2.04 times greater odds. Exclusive RYO smokers did not differ from those who smoked both RYO + FM in the odds of purchasing cheaper out-of-country cigarettes (OR=0.84; 95% CI: 0.48–1.49; p=0.55).

A third multivariable logistic regression model tested whether the odds of purchasing cheaper out-of-country cigarettes differed by country and residential location (Table 5). In this model, residential location was dichotomized to compare the effect of living in areas bordering lower-priced countries against all other areas due to small sample sizes in some cells (Table 3). Overall, the country × residential location interaction was statistically significant (Wald F=2.75; df=5, 459; p=0.018), even after controlling for sociodemographic characteristics and smoking behaviors.

**Table 5:** Adjusted per cent of smokers purchasing cheaper cigarettes outside their country of residence by country and residential location (n=5928)

*Country*	*Border–lower price*		*Otherwise*		*Difference*		*Odds Ratio*	
	*%*	*(95% CI)*	*%*	*(95% CI)*	*%*	*(SE)*	*OR*	*(95% CI)*
Germany	32.4	(22.8–43.8)	10.2	(7.3–14.1)	22.2†	(5.5)	4.53	(2.47–8.32)
Greece	21.8	(8.6–45.3)	3.4	(2.3–5.1)	18.4	(9.4)	8.35	(2.40–29.03)
Hungary	0.8	(0.1–5.6)	0.8	(0.4–1.9)	0.0	(0.8)	1.02	(0.13–8.05)
Poland	13.1	(5.7–27.5)	0.6	(0.2–1.9)	12.5	(5.2)	24.29	(6.12–96.42)
Romania	3.9	(1.4–10.8)	3.8	(1.9–7.6)	0.1	(2.4)	1.03	(0.28–3.74)
Spain	6.9	(4.5–10.4)	1.2	(0.8–2.0)	5.7†	(1.5)	6.09	(3.12–11.87)

† p<0.01 after controlling for multiple comparisons (Bonferroni correction) controlling for sex, age group, income, education, degree of urbanization, employment status, cigarettes smoked/day, time to first cigarette, smokes RYO, and plans to quit smoking.

Table 5 compares the odds of purchasing cheaper out-of-country cigarettes by country and residential location. Overall, smokers living in lower-priced border regions from Germany (OR=4.53), Greece (OR=8.35), Poland (OR=24.29), and Spain (OR=6.09) had significantly greater odds of purchasing cheaper out-of-country cigarettes compared to smokers from other areas of these countries. Table 5 also presents the adjusted percentage of smokers purchasing cheaper out-of-country cigarettes. Thirty-two per cent of German smokers and 22% of Greek smokers living in areas bordering lower-priced countries purchased cheaper cigarettes. This compares to only 10.2% of German and 3.4% of Greek smokers living in other areas of those countries. Only the difference in Germany remained statistically significant following a Bonferroni correction.

While only 7% of Spanish smokers living in areas bordering lower-priced countries purchased cheaper out-of-country cigarettes compared to 1% of Spanish smokers living in other areas, the marginal difference in adjusted prevalence remained statistically significant even after accounting for multiple comparisons (Bonferroni p<0.001). No such statistical significance was observed among Polish smokers, even though the marginal difference in that country was larger than in Spain. In Hungary and Romania, a similar percentage of smokers purchased cheaper out-of-country cigarettes irrespective of residential location.

### Prices paid per pack

Two linear regression models tested differences in the average price paid per pack by country and (a) purchase of cheaper out-of-country cigarettes and (b) use of RYO cigarettes (Table 6). In this table, exclusive RYO smokers and smokers of both RYO + FM were combined into a single category and contrasted against exclusive FM smokers. Significant interaction effects were observed after controlling for sociodemographic characteristics and smoking behaviors [(a) p=0.022, (b) p<0.001]. German smokers who purchased cheaper out-of-country cigarettes paid €0.67 less per pack than German smokers who did not purchase outside the country (Bonferroni p=0.005). No significant differences were observed among smokers from the other countries.

**Table 6:** Adjusted* self-reported cigarettes prices (€/pack of 20) by country, purchase of cheaper out-of-country cigarettes, or use of RYO cigarettes (n=5637)

*Country*	*Cheaper*			*Otherwise*			*Difference*			
	*(n)*	*Mean*	*(SE)*	*(n)*	*Mean*	*(SE)*	*Mean*	*(SE)*	*T*	*p†*
Germany	(139)	4.21	(0.19)	(795)	4.88	(0.07)	-0.67	(0.20)	-3.37	0.005
Greece	(47)	4.15	(0.26)	(916)	3.81	(0.05)	0.34	(0.26)	1.29	1.000
Hungary	(8)	3.32	(0.35)	(907)	2.89	(0.06)	0.44	(0.36)	1.23	1.000
Poland	(14)	2.6	(0.17)	(900)	2.81	(0.12)	-0.21	(0.19)	-1.10	1.000
Romania	(29)	2.73	(0.15)	(948)	2.93	(0.04)	-0.20	(0.16)	-1.29	1.000
Spain	(16)	3.51	(0.24)	(918)	3.94	(0.04)	-0.43	(0.25)	-1.68	0.560

	***Any RYO***			***Exclusive FM***			***Difference***			
	***(n)***	***Mean***	***(SE)***	***(n)***	***Mean***	***(SE)***	***Mean***	***(SE)***	***T***	***p†***

Germany	(238)	3.60	(0.15)	(696)	5.37	(0.05)	-1.76	(0.15)	-11.77	<0.001
Greece	(252)	3.64	(0.13)	(711)	3.75	(0.03)	-0.11	(0.13)	-0.84	1.000
Hungary	(465)	1.63	(0.08)	(450)	3.45	(0.05)	-1.82	(0.07)	-25.25	<0.001
Poland	(162)	3.29	(0.59)	(752)	2.88	(0.07)	0.41	(0.60)	0.68	1.000
Romania	(46)	2.98	(0.16)	(931)	3.17	(0.03)	-0.19	(0.15)	-1.26	1.000
Spain	(229)	3.07	(0.14)	(705)	4.24	(0.03)	-1.17	(0.15)	-7.88	<0.001

* Model-based average prices (marginal means) are adjusted for sex, age, income, education, degree of urbanization, employment status, cigarettes smoked per day, time to first cigarette, intentions to quit smoking and smokes RYO (for the purchase of cheaper out-of-country cigarettes model only).

† p-values are adjusted for multiple comparisons (Bonferroni correction).

Although Hungarian smokers who purchased cheaper cigarettes outside Hungary reported paying more for their purchase compared to smokers who did not, it cannot be determined from these data whether the price these smokers paid for their last purchase was for a purchase of cheaper cigarettes. In spite of that, it is worth noting that RYO use is prevalent among Hungarian smokers. When prices were estimated by country and RYO status, RYO smokers from Hungary paid significantly less per pack than exclusive FM smokers (€1.63/pack vs €3.45/pack, respectively; Bonferroni p<0.001). RYO smokers from Germany and Spain also paid significantly less per pack than exclusive FM smokers (Bonferroni p<0.001).

## DISCUSSION

While the total amount of cigarettes purchased outside the country may be minimal in the six countries studied, a minority of smokers in these countries is sensitive to prices and choose to purchase cheaper cigarettes outside the home country at least some of the time. Overall, 15% of German smokers reported purchasing cheaper out-of-country cigarettes in the previous six months while 32% of German smokers living in areas bordering lower-cost countries made such purchases compared to only 10% of German smokers living in other regions. Regression adjusted prevalence estimates showed a similar difference in Greece and a slightly smaller effect in Poland, although these differences were not statistically significant. Spanish smokers rarely purchased cheaper out-of-country cigarettes. However, Spanish smokers from the region bordering Andorra were significantly more likely to make such purchases compared to smokers from other regions of Spain (6.9% vs 1.2%, respectively). Across all countries, smokers living in regions bordering lower-cost countries had significantly higher odds of purchasing cheaper out-of-country cigarettes.

While only a minority of smokers in these countries purchased cheaper cigarettes outside their country, these findings support the hypothesis that accessibility to lower-cost alternatives provides price-sensitive smokers with incentives to make such purchases. First, in Germany, the direction of effects indicates that smokers from regions bordering Austria, the Czech Republic and Poland are more likely to purchase cheaper out-of-country cigarettes than smokers living in regions bordering Denmark, the Netherlands, Belgium, Luxembourg, France, and Switzerland.

Second, even though Spain only borders Portugal, France, and Andorra, smokers living in the region bordering Andorra were significantly more likely to purchase cheaper out-of-country cigarettes than smokers from other regions. Although Andorra is not an EU MS, the MPPC pack price in that country in 2016 was €3.50, compared to €4.85 in Spain. It is plausible that Spanish smokers living near Andorra could be drawn to that country because of its popularity as a tourist destination and duty-free shopping^25^.

Third, almost no Hungarian smokers reported purchasing cheaper out-of-country cigarettes, irrespective of where they lived. With the exception of Serbia, cigarette prices in all neighboring countries were similar or higher than in Hungary in 2016. Furthermore, more than 50% of Hungarian smokers smoke RYO cigarettes. Thus, price-sensitive Hungarian smokers can find cheaper cigarettes within their own country. Indeed, they appear to do so because RYO smokers pay, on average, €1.82 less per standardized pack than exclusive FM smokers.

Finally, these results are broadly consistent with previous studies examining cross-border purchasing in other European countries. Agaku et al.^7^ estimated that cross-border purchasing of cheaper cigarettes was highest in France, Austria, Finland, and Germany. In that study, 29% of German smokers purchased cheaper out-of-country cigarettes in the previous 12 months. Nagelhout et al.^6^ report a gradient in cross-border purchasing by residential location: 24% of French smokers and 13% of German smokers living in regions bordering lower-cost countries frequently purchased out-of-country cigarettes in the previous six months compared to only 5% of smokers living in non-border areas in either country. A similar effect was observed among German smokers in the current study.

In this study, daily cigarette consumption was significantly associated with purchasing cheaper out-of-country cigarettes. Heavier smokers had significantly greater odds of making such purchases than smokers consuming ≤10 cigarettes/day. Neither time to first cigarette of the day nor heaviness of smoking were significantly associated with purchasing cheaper out-of-country cigarettes. While CPD, TTF and HSI all measure some aspect of nicotine dependence, it is striking that only CPD was associated with purchasing cheaper out-of-country cigarettes. Since CPD is directly tied to smokers’ immediate financial costs for cigarettes, it is a better indicator of price sensitivity than TTF or HSI.

Unlike previous research^6,9,10^, low income smokers were not significantly more likely to purchase cheaper out-of-country cigarettes than high income smokers (p=0.217). The lack of an effect suggests household income may be less important than direct measures of financial cost, such as CPD, in smokers’ decisions to purchase cheaper out-of-country cigarettes.

### Strengths and limitations

A strength of this study was that it used a more granular classification of residential location than Nagelhout et al.^6^ for a different set of European countries. In so doing, it was possible to identify a clear relationship between potential accessibility to lower-cost cigarettes and the likelihood of purchasing cheaper out-of-country cigarettes. Even so, it was not possible to identify the country from which smokers purchased cheaper cigarettes or whether smokers made special trips to a lower-cost country to purchase cigarettes or smokers were already visiting a lower-cost country when they made their purchase. Furthermore, data were collected between June and September 2016 during the peak holiday season in Europe, and the reference period, namely the previous six months, depended on interview date. This may have introduced some bias, since some smokers able to take their vacation in other countries may have purchased cheaper cigarettes because they were already visiting a lower-cost country. Therefore, more nuanced approaches incorporating theoretical perspectives developed in the field of time geography could reveal when and where smokers spend their time and for what purposes^26^. This approach permits identification of purchase locations and a more complete assessment of whether tax harmonization policies exert their intended effects.

The method used to classify smokers’ residential location is subject to possible misclassification. In particular, NUTS regions were classified as border regions using the distance between a region’s geographic centroid and the border of the nearest neighboring country. For regions bordering two countries, a region could have been classified as bordering a higher-cost country even though smokers could easily travel to the lower-cost country. Moreover, the methods used here do not account for whether additional travel costs were offset by the purchase of cheaper cigarettes. Although the classification method was crude, strong effects of residential location on the likelihood of purchasing cheaper out-of-country cigarettes were observed in areas where they would be expected. More nuanced methods could assess smokers’ specific travel patterns to identify when and where smokers make out-ofcountry purchases.

Another limitation is that cheaper out-of-country purchases were relatively rare in five of the six countries studied. As a result, prevalence estimates of cross-border purchasing are based on relatively few smokers. These estimates may therefore be unstable and must be interpreted cautiously. However, the effect of residential location pooled across all six countries suggests that potential access to cheaper out-of-country cigarettes is associated with greater odds of making such purchases. Although this finding could have been driven by the effect among German smokers alone, a country by residential location interaction effect points to an effect of living near lower-priced jurisdictions in some of the other countries, particularly in Spain.

In some of the countries studied, a smaller price differential might influence price-sensitive smokers to purchase cheaper out-of-country cigarettes. Although a €1 difference in the MPPC pack price was used to identify lower-cost jurisdictions, a relative price difference of only 10% might provide sufficient incentives to purchase cheaper out-of-country cigarettes. A sensitivity analysis using this relative price difference had negligible effects on the results reported here.

Finally, price differences between exclusive RYO smokers and FM smokers may have been influenced by the factor used to convert the amount of RYO tobacco purchased to cigarette equivalents (0.75 g equivalent). Moreover, a single conversion factor was assumed for all countries in this study, even though conversion factors might vary across countries^16^. However, the purpose of converting all self-reported prices to standardized pack prices, irrespective of exclusive RYO use, was to demonstrate that smokers who purchased out-of-country cigarettes paid, on average, less than smokers who did not. Differences in self-reported prices were also in the expected direction for RYO smokers compared to FM smokers.

## CONCLUSIONS

As of 2016, price-sensitive smokers in select European countries have access to, and purchase cheaper cigarettes from, other European countries having MPPC pack prices at least €1 lower than in smokers’ home countries. This is particularly important in Germany. In 2016, the MPPC price of a pack of cigarettes in Germany was €6 while it was €5 in Austria, €3.38 in Poland, and €3.11 in the Czech Republic. These price differentials provide incentives for price-sensitive German smokers to purchase cigarettes outside their country. Tax harmonization policies that minimize such differentials can eliminate sources of lower-cost alternatives. Indeed, the EU has enacted such policies (Council Directive 2011/64/ EU), although new MS had until 31 December 2017 to comply^3,11^. In spite of this directive, López-Nicolás and Stoklosa^3^ found that differentials in cigarette prices remained stable between EU MS since 2004. Thus, future research should continue to estimate the prevalence of cross-border purchasing in Europe to evaluate whether taxation policies effectively equalize prices across jurisdictions thereby eliminating lower-cost alternatives for price-sensitive smokers. Prices should also be equalized across products (i.e. RYO vs FM cigarettes) to discourage smokers from switching to lower-cost alternatives^3,27^. In conjunction with such policies, price-sensitive smokers should be encouraged to quit smoking rather than seek out lower-cost alternatives.

## CONFLICTS OF INTEREST

The authors declare that they have no competing interests, financial or otherwise, related to the current work. K Przewoźniak reports grants and personal fees from the Polish League Against Cancer, outside the submitted work. CI Vardavas reports that he is the Strategic Development Editor of TID and that there are no conflicts of interest with this current work. The rest of the authors have also completed and submitted an ICMJE form for disclosure of potential conflicts of interest.
