# Supplementary material for: Cross-border purchasing of cigarettes among smokers in Six Countries of the EUREST-PLUS ITC Europe Surveys
Source: Tob Induc Dis. 2019 Mar 7;16:A13. doi: 10.18332/tid/100411 (PMC6661845; doi:10.18332/tid/100411)
Supplement: Supplementary file 1 [file TID-16-A13-s1.pdf]

Supplementary Table 1. Price of cigarettes in the most popular price category (MPPC) in countries bordering NUTS2 and NUTS3 regions of countries participating in the ITC Six European Country Survey, Wave 1.

Country (MPPC)	Region	Neighbours	EUMS	MPPC Price
Germany (n = 1003)	Non-border	—	—	—
(6.00)	Border, similar/higher	Denmark	Y	5.92
		Netherlands	Y	6.63
		Belgium	Y	6.32
		Luxembourg	Y	5.20
		France	Y	7.00
		Switzerland	N	7.85
	Border, lower	Austria	Y	5.00
		Czech Republic	Y	3.11
		Poland	Y	3.38
Greece (n = 1000)	Non-border	—	—	—
(4.00)	Border, similar/higher	Turkey	N	4.04
	Border, lower	Bulgaria	Y	2.51
		Macedonia	N	1.08
		Albania	N	1.69
Hungary (n = 1000)	Non-border	—	—	—
(3.70)	Border, similar/higher	Slovakia	Y	3.10
		Austria	Y	5.00
		Slovenia	N	3.50
		Croatia	Y	3.23
		Romania	Y	3.60
	Border, lower	Serbia	N	1.78
		Ukraine	N	0.53
Poland (n = 1006)	Non-border	—	—	—
(3.38)	Border, similar/higher	Germany	Y	6.00
		Czech Republic	Y	3.11
		Slovakia	Y	3.10
		Lithuania	Y	3.10
	Border, lower	Ukraine	N	0.53
		Russia	N	1.42
Romania (n = 1001)	Non-border	—	—	—
(3.60)	Border, similar/higher	Hungary	Y	3.70
	Border, lower	Ukraine	N	0.53
		Serbia	N	1.78
		Bulgaria	Y	2.51
		Moldova	N	0.91
Spain (n = 1001)	Non-border	—	—	—
(4.85)	Border, similar/higher	Portugal	Y	4.80
		France	Y	7.00
	Border, lower	Andorra	N	3.50

EUMS = European Union Member State.

Supplementary Table 2. Classification of monthly household income.

Country	Low	Moderate	High
Germany	< €1750	€1750 to < €3000	≥ €3000
Greece	< €750	€750 to < €1500	≥ €1500
Hungary	≤ 150,000 Ft	150,001 Ft to ≤ 250,000 Ft	> 250,000 Ft
Poland	≤ 2000 zl	2001 zl to 4000 zl	> 4000 zl
Romania	≤ 1000 lei	1001 lei to 2500 lei	> 2500 lei
Spain	< €1250	€1250 to < €2500	≥ €2500

Supplementary Table 3. Frequency of any out-of-country purchase among smokers participating in Wave 1 of the ITC Six European Country Survey by country of residence (weighted estimates).

	DE			GR			HU			PL			RO			ES		
	(n)	%	(95% CI)	(n)	%	(95% CI)	(n)	%	(95% CI)	(n)	%	(95% CI)	(n)	%	(95% CI)	(n)	%	(95% CI)
Any purchase outside country																		
Never	(792)	79.2	(75.3, 82.6)	(917)	90.6	(86.4, 93.6)	(975)	97.7	(96.4, 98.6)	(935)	92.2	(89.7, 94.2)	(890)	87.0	(82.6, 90.4)	(948)	95.5	(94.0, 96.7)
Only once	(85)	8.9	(6.8, 11.6)	(32)	3.0	(2.0, 4.3)	(12)	1.1	(0.5, 2.1)	(22)	2.4	(1.5, 3.6)	(18)	2.4	(1.2, 4.2)	(26)	2.0	(1.2, 3.1)
A few times	(86)	7.9	(6.1, 10.1)	(36)	4.8	(2.1, 9.1)	(11)	1.0	(0.5, 1.8)	(25)	3.2	(1.8, 5.1)	(35)	4.3	(2.6, 6.6)	(21)	2.0	(1.0, 3.5)
Many times	(14)	1.6	(0.7, 3.0)	(11)	1.3	(0.6, 2.2)	(2)	0.2	(0.0, 0.7)	(20)	2.2	(1.3, 3.6)	(40)	4.7	(3.1, 6.8)	(6)	0.5	(0.2, 1.2)
All the time	(25)	2.5	(1.0, 5.0)	(4)	0.3	(0.1, 0.9)	(0)	–	–	(0)	–	–	(15)	1.6	(0.8, 2.8)	(0)	–	–

© 2019 Driezen P.
